# The course of untreated anxiety and depression, and determinants of poor one-year outcome: a one-year cohort study

**DOI:** 10.1186/1471-244X-10-86

**Published:** 2010-10-20

**Authors:** Ilse MJ van Beljouw, Peter FM Verhaak, Pim Cuijpers, Harm WJ van Marwijk, Brenda WJH Penninx

**Affiliations:** 1 Netherlands Institute for Health Services Research, Utrecht, the Netherlands; 2 Department of Clinical Psychology, VU University, Amsterdam, the Netherlands; 3 Department of General Practice, VU University Medical Centre, Amsterdam, the Netherlands; 4 Department of Psychiatry/EMGO Institute, VU University Medical Centre, Amsterdam, the Netherlands; 5 Department of Psychiatry, Leiden University Medical Center, Leiden, the Netherlands; 6 Department of Psychiatry, University Medical Centre Groningen, University of Groningen, Groningen, the Netherlands

## Abstract

**Background:**

Little is known about the course and outcome of untreated anxiety and depression in patients with and without a self-perceived need for care. The aim of the present study was to examine the one-year course of untreated anxiety and depression, and to determine predictors of a poor outcome.

**Method:**

Baseline and one-year follow-up data were used of 594 primary care patients with current anxiety or depressive disorders at baseline (established by the Composite Interview Diagnostic Instrument (CIDI)), from the Netherlands Study of Depression and Anxiety (NESDA). Receipt of and need for care were assessed by the Perceived Need for Care Questionnaire (PNCQ).

**Results:**

In depression, treated and untreated patients with a perceived treatment need showed more rapid symptom decline but greater symptom severity at follow-up than untreated patients without a self-perceived mental problem or treatment need. A lower education level, lower income, unemployment, loneliness, less social support, perceived need for care, number of somatic disorders, a comorbid anxiety and depressive disorder and symptom severity at baseline predicted a poorer outcome in both anxiety and depression. When all variables were considered at the same time, only baseline symptom severity appeared to predict a poorer outcome in anxiety. In depression, a poorer outcome was also predicted by more loneliness and a comorbid anxiety and depressive disorder.

**Conclusion:**

In clinical practice, special attention should be paid to exploring the need for care among possible risk groups (e.g. low social economic status, low social support), and support them in making an informed decision on whether or not to seek treatment.

## Background

Anxiety and depression have serious consequences for patients, their family, and for society. However, many mental disorders remain untreated [1-8]. In general, undetected and untreated patients have less severe symptoms than detected patients who receive treatment [9-12].

It is important to take patients' preferences and views into account. Some patients can find a way to deal with their symptoms. There even are patients who do not perceive a mental problem, despite fulfilling the criteria for a CIDI-diagnosis of anxiety or depression, or who simply do not perceive a need for care [13,14].

In Moitabai's study [1], one third of untreated patients reported unmet needs, especially younger patients, higher educated patients and patients with insurance problems. In our own study [13], based on baseline data from the Netherlands Study of Depression and Anxiety (NESDA), we found that 25% of untreated patients with a current anxiety and/or depressive disorder perceived themselves as mentally healthy. Twenty-six percent had no perceived need for care, and 49% perceived a need for care which was not met, especially in patients from ethnic minority groups and patients with a lack of social support. It was found that subjects with an unmet perceived need for care reported equally severe and clinically relevant symptoms at baseline as subjects who received professional care. Patients without a perceived need had less symptoms than patient with a met or unmet need. This has been found in other studies as well [15].

It becomes problematic when untreated patients have a worse outcome than would be the case if they were treated. Rost et al. [16] found that undetected and untreated patients with major depression in primary care have poor outcomes compared with treated patients. In this study, however, untreated patients were followed up, regardless of their own perceived need for treatment.

To our knowledge, outcome of untreated anxiety and depression in patients with and without a self-perceived need for care has not yet been studied. As self perceived need for care might be an important modifier for the risk of not being treated, we will include this parameter while searching for consequences of not being treated and for determinants of possible poor outcome after not being treated.

### Aims of the study

The aim of this study was to investigate the consequences of being untreated for an anxiety or depressive disorder at one-year follow-up, in patients with and without a need for care. In addition, determinants of a poor outcome in untreated patients were evaluated.

## Methods

### Sampling and data collection

All data used in this study were derived from the Netherlands Study of Depression and Anxiety (NESDA). NESDA is a multi-site naturalistic study, and aims at studying the long-term course and consequences of anxiety and depressive disorders for a period of eight years. The analyses presented in this study are based on the baseline (2004-2006) and one-year follow-up assessment. Procedures of NESDA are described in detail elsewhere [17]. The study protocol was approved centrally by the Ethics Review Board of the VU University Medical Centre, and subsequently by local review boards of each participating center.

**Figure 1:**
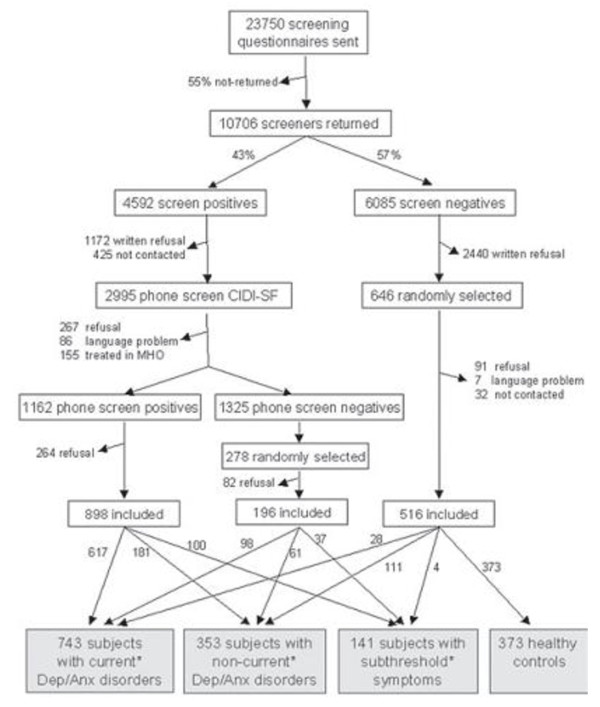
**Recruitment flow of NESDA-respondents in the primary care sample**. *Current = presence during the last six month; non-current = presence before the last six months; subthreshold symptoms are defined as screen-postives of having a minor depression according to the CIDI-interview.

In brief, respondents were recruited from 65 general practitioners (GPs) in the vicinity of the field sites (Amsterdam, Leiden, Groningen) using a three-stage procedure (see figure 1). Firstly, a random selection of 23,750 patients aged 18 to 65 years who consulted their GP in the last four months - irrespective of the reason for their visit - were sent a Kessler-10 screening questionnaire [18], measuring psychological distress, and five additional anxiety questions. The response rate was 45% (N = 10,706). Of this group, the 4,592 screen-positives were additionally screened during a brief telephone interview conducted by trained research staff, consisting of a short form of the Composite Interview Diagnostic Instrument (CIDI) [19]. Ultimately, 743 respondents who met the criteria for a six-month anxiety or depressive disorder (established by a full CIDI, and including a major depressive disorder, dysthymia, general anxiety disorder, social phobia, panic disorder or agoraphobia), and who were fluent in Dutch were included for the baseline assessment (T0). Of these, 594 respondents (79.9%) participated in the one-year follow-up assessment (T1).

### Measures

#### Dependent variables

The dependent variables used in this study are severity of depression and anxiety at baseline and one-year follow-up, measured by the 30-item Inventory of Depressive Symptomatology (Self-Report; IDS-SR) [20] and the 21-item Beck Anxiety Inventory (BAI) [21], respectively.

#### Independent variables

All determinants used in this paper were addressed at T0.

##### Determinants of a poor clinical outcome

Determinants of outcome are classified according to Andersen's behavioral model [22,23], and include: 1) *predisposing *factors such as socio-demographic characteristics; 2) factors that *enable *the use of services such as income; and 3) factors that determine the *need for care.*

***Predisposing factors:*** Information was gathered concerning socio-demographic characteristics such as age, gender, education level, country of birth, marital status and household composition. Social support was addressed by the number of family members, friends and acquaintances (adults only, household members excluded) with whom the respondent reported to be in regular and important contact. The De Jong-Gierveld Loneliness Scale [24] measures the amount of loneliness a respondent experiences by citing 11 statements such as 'I often feel rejected', which can be rated on a 3-point Likert scale.

***Enabling factors:*** The income level and employment status of the respondent were ascertained during the interview.

***Need for care:*** Two types of need for care were distinguished: a subjective and an objective need for care. A subjective need for mental health care is perceived by the patient and was ascertained by the Perceived Need for Care Questionnaire (PNCQ) [25]. The PNCQ is a fully structured interview that assesses the patient's perception of the presence of a mental problem, the perceived need for care and the patient's utilization of health care services. This translates as whether the patient consulted a GP, specialist, company doctor, social worker, psychologist, psychiatrist, psychotherapist or mental health institution for a mental problem. Patients who confirmed contact with at least one health care provider about a mental health problem were considered 'treated'. Patients who did not, were considered 'untreated'.

Patients' self-reported perceived need for care, was assessed for six types of care: information, medication, counseling, practical support, skills training and referral to a mental health care specialist. For each domain, respondents indicated if care was received (met need) and, if not, if care was wanted (unmet need) or not (no need). The PNCQ has shown acceptable reliability and validity for use in a community sample [25]. Although the Dutch version of the PNCQ has not specifically been validated, a study comparing PNCQ data from an Australian and a Dutch sample of primary care patients with anxiety and/or depression, showed many similarities between the given answers [26].

By means of the PNCQ, three patient groups with a DSM-IV diagnosis of anxiety or depression were distinguished, based on various reasons for not receiving treatment: 1) untreated patients who did not perceive themselves as having a mental problem; 2) untreated patients who perceived themselves as having a mental problem, but who did not report any need for care; and 3) untreated patients who perceived themselves as having a mental problem and expressed a need for care. These three groups will be compared with 4) patients with a DSM-IV diagnosis who received treatment.

An objective or clinical need for care is indicated by symptom severity (measured by the IDS and BAI), the presence of a comorbid anxiety or depressive disorder, a single or a recurrent disorder in case of a depression, and the recency of the experienced symptoms (measured by the CIDI). When multiple anxiety and/or depressive disorders where diagnosed, the symptom duration of the less recent disorder was used. To create an index of somatic health, an inventory was constructed to assess the number of chronic somatic diseases for which medical treatment was received.

### Statistical analysis

Firstly, we explored potential differences between completers and non-completers of the one-year follow-up assessment in NESDA, by using χ^2 ^analyses (for categorical variables) and t-tests (for continuous variables).

Secondly, we examined the one-year course of anxiety and depression in untreated and treated patients separately by performing multilevel repeated measures ANCOVA's, using baseline and one-year follow-up scale scores of symptom severity in anxiety (BAI) and depression (IDS), respectively. The previous mentioned predisposing, enabling and need for care factors were added as covariates. To take into account the possible influence of GPs on the patients' treatment receipt, multilevel models with random intercepts were used, consisting of patients (level 1) nested within GPs (level 2). Specifically, in the multilevel repeated measures ANCOVA's, chi-squared tests were performed to compare the regression weights of the course of anxiety and depression in each patient group, controlling for the influence of different predisposing, enabling and need for care factors. Multilevel modeling takes into account all available baseline and one-year follow-up data from both completers and non-completers, and imputes missing data from respondents who completed only the baseline assessment.

Furthermore, to determine the characteristics of clinical outcome at T1, multilevel univariate linear regression analyses with random intercepts were performed for anxiety (using the BAI scale scores at T1) and depression (using the IDS scale scores at T1) separately. Additionally, a multilevel multivariate linear regression model with random intercepts was used to determine which of the previously mentioned characteristics predicted clinical outcome when all variables were considered simultaneously. Baseline scale scores of the BAI and IDS were added to control for baseline symptom severity. Since these analyses aimed at predicting clinical outcome at T1, we were unable to impute missing data. Therefore, only respondents who completed the one-year follow-up assessment were considered in these analyses. The multilevel repeated measures ANCOVA's were carried out in MLwiN 2.02; for all other analyses, STATA 10.0 was used.

## Results

### Characteristics of the study sample

The sample contains 594 respondents, and 71.2% are women (N = 423). At baseline, respondents were on average 45.7 years old (sd. 11.9 years), with the youngest participant being 18 years of age and the oldest 65. Participants had an average of 12.0 years (sd. 3.4 years) of education, ranging from 5 to 18 years. The majority of patients had a six-month diagnosis for an anxiety disorder (79.1%; N = 470); 56.2% (N = 334) were diagnosed with a depression, and 35.4% (N = 210) of patients suffered from both.

Compared to baseline assessment, 20.1% (N = 149) of the respondents were lost to attrition at one-year follow-up. Compared to non-completers, completers were older (45.7 vs. 41.6; p < .01), had a higher level of education (p < .01), experienced more loneliness (5.1 vs. 3.0; p < .001) and social support (6.7 vs. 5.6; p < .05), and reported less severe symptoms of anxiety (15.2 vs. 19.4; p < .001) and depression (26.5 vs. 29.8; p < .01). A description of the untreated and treated patients is given in Table 1.

**Table 1:** Differences between untreated and treated patients at T0 (N = 594)

	1. Untreated - unperceived problem		2. Untreated - unperceived need		3. Untreated - unmet perceived need		4. Treated	
	M ± SD/%	N	M ± SD/%	N	M ± SD/%	N	M ± SD/%	N
*Predisposing characteristics*								
Male gender (%)	34.8	24	30.0	21	29.0	36	27.2	90
Age (%)								
1. 18-35	21.7	15	12.9	9	22.6	28	26.6	88
2. 36-50	27.5	19	34.3	24	32.3	40	36.3	120
3. 51-65	50.7	35	52.9	37	45.2	56	37.2	123
Education (%)								
1. Basic	5.8	4	7.1	5	13.7	17	5.7	19
2. Intermediate	60.9	42	62.9	44	53.2	66	59.5	197
3. High	33.3	23	30.0	21	33.1	41	34.7	115
Born outside the Netherlands (%)^a^*	13.0	9	5.7^3^	4	18.6^2,4^	23	9.4^3^	31
Marital status (%)								
Never married	40.6	28	32.9	23	36.3	45	42.0	139
Currently married	42.0	29	45.7	32	46.8	58	40.2	133
Formerly married	17.4	12	21.4	15	16.9	21	17.8	59
Household composition - alone (%)	33.3	23	37.1	26	33.9	42	33.8	112
Loneliness (M ± SD; range 0-10)^b^***	3.5 ± 3.2^3,4^	68	4.6 ± 3.7^3^	69	6.2 ± 3.7^1,2^	123	5.3 ± 3.8^1^	328
Social support (M ± SD; range 0-22)^c^**	8.4 ± 5.6^3^	69	7.3 ± 5.2	70	5.5 ± 4.3^1^	124	6.6 ± 5.1	331
*Enabling factors*								
Income in euro's p.m.(%)								
< € 2.400,-	60.9	42	62.7	42	68.9	84	63.0	208
> € 2.400,-	39.1	27	37.3	25	40.3	50	30.8	102
Employment status - unemployed (%)	29.0	20	38.6	27	40.3	50	30.8	102
*Need factors*								
Type of disorder								
Major depression single (%)^d ^***	7.3 ^3,4^	5	8.6 ^3,4^	6	21.8 ^1,2^	27	28.7 ^1,2^	95
Major depression recurrent (%)^e ^***	5.8 ^2,3,4^	4	28.6 ^1^	20	31.5 ^1^	39	37.5 ^1^	124
Dysthemia (%)^f ^**	4.4 ^3,4^	3	7.1 ^3,4^	5	17.7 ^1,2^	22	17.8 ^1,2^	59
General anxiety disorder (GAD) (%)^g ^**	10.1 ^3,4^	7	14.3 ^4^	10	25.8 ^1^	32	29.3 ^1,2^	97
Social phobia (%)^h ^*	39.1	27	24.3 ^3,4^	17	46.0 ^2^	57	37.2 ^2^	123
Panic without agoraphobia (%)^I ^**	10.2	7	27.1 ^3,4^	19	12.9 ^2^	16	11.8 ^2^	39
Panic with agoraphobia (%)	15.9	11	15.7	11	16.1	20	24.8	82
Agoraphobia without panic (%)	21.7	15	17.1	12	16.9	21	11.2	37
At least one depressive disorder (%)^j ^***	14.5 ^2,3,4^	10	38.6 ^1,3,4^	27	56.5 ^1,2,4^	70	68.6 ^1,2,3^	227
Comorbid anxiety and depressive disorder (%)^k^***	1.5 ^2,3,4^	1	20.0 ^1,3,4^	14	37.9 ^1,2^	47	44.7 ^1,2^	148
Recency (%)								
<6 months	46.4	32	41.4	29	51.6	64	56.5	187
6 - 12 months	4.4	3	4.3	3	6.5	8	6.0	20
>12 months	49.3	34	54.3	38	41.9	52	37.5	124
Number of somatic diseases (M ± SD)	.8 ± 1.0	69	.5 ± 1.0	70	.9 ± 1.1	124	.7 ± 1.1	331
Severity anxiety (BAI) T0 (M ± SD; range 0-63)^l^***	8.2 ± 5.2^2,3,4^	69	12.4 ± 8.0^1,3,4^	70	16.5 ± 9.3^1,2^	124	16.7 ± 9.8^1,2^	331
Severity of depression (IDS) (M ± SD; range 0-84)^m^***	15.7 ± 7.3^2,3,4^	68	22.0 ± 8.4^1,3,4^	70	28.6 ± 9.5^1,2^	124	29.0 ± 11.4^1,2^	330

* p < .05 **p < .01 *** p < .001

^1,2,3,4 ^numbers refer to groups who differ significantly from each other

^a ^χ ^2^(3) = 10.14, p = .017

^b ^F(3,584) = 8.64, p = .000

^c ^F(3,590) = 5.03, p = .002

^d ^χ^2^(3) = 24.41, p = .000

^e ^χ^2^(3) = 26.87, p = .000

^f ^χ^2^(3) = 12.34, p = .006

^g ^χ^2^(3) = 15.84, p = .001

^h ^χ^2^(3) = 9.07, p = .028

^i ^χ^2^(3) = 12.58, p = .006

^j^χ^2^(3) = 78.22, p = .000

^k^χ^2^(3) = 54.96, p = .000

^l ^F(3,590) = 20.02, p = .000

^m ^F(3,588) = 38.03, p = .000

### The course of depression and anxiety

**Figure 2:**
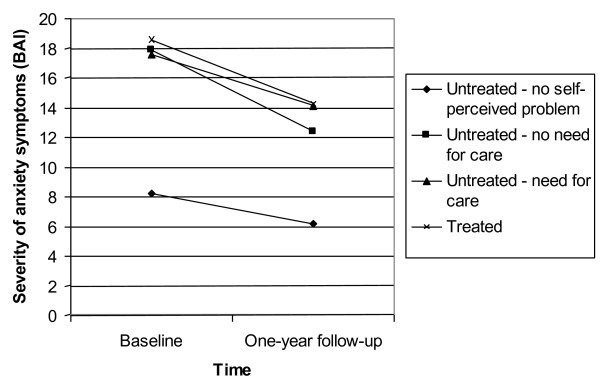
**The course of anxiety in patients with a CIDI-diagnosis of an anxiety disorder at T0, in the treatment/non-treatment groups (N = 422) (range: 0-63)**. Data of respondents who did not complete the one-year follow-up assessment were also included in the multilevel repeated measures ANCOVA.

**Figure 3:**
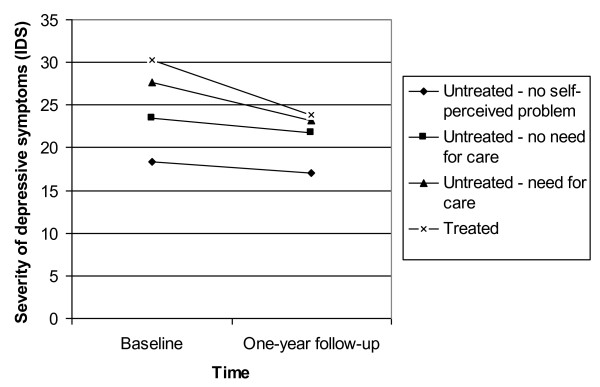
**The course of depression in patients with a CIDI-diagnosis of a depressive disorder at T0, in the treatment/non-treatment groups (N = 573) (range: 0-84)**. Data of respondents who did not complete the one-year follow-up assessment were also included in the multilevel repeated measures ANCOVA.

Figures 2 and 3 show the results of the multilevel repeated measures ANCOVA's, examining the course of anxiety measured by the BAI, and the course of depression assessed by the IDS, at T0 and T1. All patients suffered from a CIDI-diagnosis of anxiety or depression, respectively. Data of respondents who completed only the baseline assessment were also taken into account.

The course of depression differs between untreated patients without a self-perceived mental problem compared to untreated patients with an unmet need for care (χ^2 ^= 6.35, p < .05) and treated patients (χ^2 ^= 22.16, p < .001). Also, untreated patients without a need for care show a different one-year course than untreated patients with an unmet need for care (χ^2 ^= 4.25, p < .05) and treated patients (χ^2 ^= 16.08, p < .001).

In anxiety, the one-year course only differs between untreated patient without a self-perceived mental problem and untreated patients without a need for care (χ^2 ^= 3.85, p < .05).

### Determinants of a poor clinical outcome

Next, risk factors of a poor clinical outcome were examined by multilevel univariate and multivariate linear regression analyses. The results are shown in Table 2 (anxiety) and 3 (depression).

**Table 2:** Potential risk factors of a poor outcome in anxiety at T1: multilevel univariate and multivariate linear regression analyses.

	Univariate			Multivariate		
		
	β	SE	Variance at GP-level (SE)	β	SE	Variance at GP-level (SE)
*Predisposing characteristics*						
Male gender	-.97	.98	.08 (2.21)	.20	.79	
Age						
1. 18-35	ref			ref		
2. 36-50	1.94	1.20		1.29	1.05	
3. 51-65	1.56	1.15	.18 (2.23)	1.48	1.11	
Education						
1. Basic	ref			ref		
2. Intermediate	-2.87	1.59		-.08	1.31	
3. High	-6.09***	1.68	1.57 (2.51)	-1.59	1.43	
Born outside the Netherlands	2.25	1.42	.00 (.01)	.60	1.14	
Marital status						
Never married	-.72	.99		.35	1.06	
Currently married	ref			ref		
Formerly married	-.11	1.25	.33 (2.29)	-1.28	1.06	
Household composition - alone	.37	.96	.08 (2.24)	.09	.97	
Loneliness (range 0-10)	.40**	.12	.00 (.00)	-.02	.10	
Social support (range 0-22)	-.21*	.09	.00 (.00)	-.05	.07	
*Enabling factors*						
Income in euro's p.m.						
< € 2.400,-	ref			ref		
> € 2.400,-	-2.86**	.92	.00 (.00)	-1.48	.86	
Employment status - unemployed	3.49***	.91	.00 (.01)	.42	.81	
*Need factors*						
Perceived need for care						
1. No need for care - no perceived mental Problem	-7.40***	1.34		-1.78	1.23	
2. No need for care - a perceived mental problem	-3.47**	1.36		-.19	1.18	
3. Need for care - unmet	.40	1.09	.16 (2.11)	.64	.93	
4. Need for care - met	ref			ref		
Recency						
<6 months	ref			ref		
6 - 12 months	3.08	2.02		2.85	1.63	
>12 months	-1.08	.91	.00 (.00)	-.04	.76	
Comorbid anxiety and depressive disorder	4.31***	.87	.00 (.00)	.16	.84	
Number of somatic diseases	1.30**	.44	.01 (1.96)	.57	.39	
Severity of anxiety T0 (BAI; range 0-63)	.59***	.04	1.47(1.68)	.54***	.04	
						1.54 (1.71)

* p < .05 **p < .01 *** p < .001

**Table 3:** Potential risk factors of a poor outcome in depression at T1: multilevel univariate and multivariate linear regression analyses.

	Univariate			Multivariate		
		
	β	SE	Variance at GP-level (SE)	β	SE	Variance at GP-level (SE)
*Predisposing characteristics*						
Male gender	-.87	1.46	.00 (.00)	-.61	1.17	
Age						
1. 18-35	ref			ref		
2. 36-50	1.08	1.74		-1.61	1.54	
3. 51-65	4.44	1.70	.00 (.00)	2.07	1.64	
Education						
1. Basic	ref			ref		
2. Intermediate	-5.65*	2.53		-1.93	2.07	
3. High	-8.16**	2.63	.00 (.00)	-3.23	2.24	
Born outside the Netherlands	4.30*	1.97	.00 (.00)	1.57	1.60	
Marital status						
Never married	-1.70	1.46		.08	1.59	
Currently married	ref			ref		
Formerly married	-.27	1.83	.00 (.00)	-1.69	1.62	
Household composition - alone	.11	1.38	.00 (.00)	-.79	1.44	
Loneliness (range 0-10)	.89***	.17	.00 (.00)	.39*	.16	
Social support (range 0-22)	-.36**	.14	1.11 (4.83)	-.02	.12	
*Enabling factors*						
Income in euro's p.m.						
< € 2.400,-	ref			ref		
> € 2.400,-	-2.79*	1.38	.00 (.00)	-1.16	1.31	
Employment status - unemployed	4.77***	1.34	.00 (.00)	.26	1.18	
*Need factors*						
Perceived need for care						
1. No need for care - no perceived mental problem	-8.00*	3.97		2.71	3.20	
2. No need for care - a perceived mental problem	-1.43	2.47		2.00	2.07	
3. Need for care - unmet	3.05	1.62		1.55	1.31	
4. Need for care - met	ref		.00 (.00)	ref		
Recurrent depressive disorder	-1.52	1.32	.00 (.00)	.79	1.07	
Recency						
<6 months	ref			ref		
6 - 12 months	1.01	2.98		-.68	2.34	
>12 months	-1.54	1.46	.00 (.00)	-.72	1.18	
Comorbid anxiety and depressive disorder	8.23***	1.29	.00 (.01)	2.95*	1.18	
Number of somatic diseases	1.78**	.54	.00 (.00)	.23	.49	
Severity of depression T0 (IDS; range 0-84)	.63***	.05	3.16 (3.71)	.53***	.05	
						2.59 (3.29)

* p < .05 **p < .01 *** p < .001

At T1, symptom severity in anxiety was negatively associated with a higher education level (β = -6.09, SE = 1.68, p < .001), social support (β = -.21, SE = .09, p < .05), a higher income (β = -2.86, SE = .92, p < .01), perceiving no mental problem (β = -7.40, SE = 1.34, p < .001) or perceiving no need for care (β = -3.47, SE = 1.36, p < .05). Positive associations were found between more symptom severity in anxiety and loneliness (β = .40, SE = .12, p < .01), being unemployed (β = 3.49, SE = .91, p < .001), suffering from a comorbid depressive disorder (β = 4.32, SE = .87, p < .001) or from somatic diseases (β = 1.30, SE = .44, p < .01) and greater symptom severity at baseline (β = .59, SE = .04, p < .001). The same associations were found in depression (see Table 3). Additionally, persons with a depressive disorder who were born outside the Netherlands were at risk of a higher symptom severity at one-year follow-up than respondents born in the Netherlands (β = 4.30, SE = 1.97, p < .05).

Furthermore, multilevel multivariate linear regression analyses were performed (see last columns of Table 2 and 3). When all variables were considered simultaneously, only baseline symptom severity predicted clinical outcome at one-year follow-up in respondents with an anxiety disorder (β = .54, SE = .04, p < .001). In depression, besides baseline symptom severity (β = .53, SE = .05, p < .001), a higher symptom severity at one-year follow-up was also predicted by more loneliness (β = .39, SE = .16, p < .05) and having a comorbid anxiety disorder (β = 2.95, SE = 1.18, p < .05).

## Discussion

Our results revealed that all groups of untreated and treated patients showed a modest decrease in anxiety and depressive symptoms after one year. Although untreated patients with a perceived need for care and treated patients showed a more rapid symptom decrease, rank order in symptom severity was maintained: they experienced more severe symptoms at T0 and T1 than untreated patients without a perceived mental problem (in anxiety or depression) or without a perceived need for care (in depression only). This association between initial severity and symptom decline at follow-up has been noted previously [27]. Furthermore, our findings confirm previous results from the NEMESIS study [28], which concluded that more intensive treatment is associated with a poorer outcome at one-year follow-up. This is clinically a logical finding as it points at confounding by indication.

Initially, we found that a poor clinical outcome in depression and anxiety was determined by a lower education level, increased loneliness, less social support, a lower income, unemployment, perceiving a need for care, the presence of a comorbid anxiety or depressive disorder, somatic diseases and increased baseline symptom severity. In depression, higher symptom severity at one-year follow-up was also predicted by being born outside the Netherlands.

Despite these findings from univariate analyses, however, only increased loneliness and the presence of a comorbid anxiety disorder maintained their significance in predicting a poor outcome in depression when controlled for baseline symptom severity. Apparently, most differences in predisposing, enabling and need factors were attributable to initial symptom severity. In anxiety, baseline symptom severity appeared to be the only predictor of a poor outcome at follow-up in the multivariate analysis. Indeed, other community studies likewise showed that symptom severity at baseline was (one of) the most prominent determinant(s) of poor outcome [27,29-31]. However, to our knowledge, the finding that increased loneliness predicts a poor outcome in depression, independently of baseline symptom severity, has not been shown before in a community sample.

Younger age appeared to be a mutually independent predictor of poor outcome in the study of Spijker et al. [29]. Differences in study design may account for the fact that this finding was not replicated by our study: Spijker et al. [29] defined severity as a severe disorder with psychotic features. Moreover, perhaps our study population differed from the population they studied: respondents who completed the one-year follow-up in the NESDA-study were, for instance, older and lonelier than non-completers, which could have affected our results.

### Strengths and weaknesses of the study

An important strength of the present study concerns the inclusion and comprehensive measurement of perceived need for care for a mental disorder, using the PNCQ. Furthermore, we made use of a large sample. However, in considering the results reported here, some limitations must be noted.

Firstly, our study employed observational data. No conclusions about a causal relationship between care utilization and clinical outcome can therefore be drawn. Our data are not suitable for determining the effectiveness of treatments. Moreover, our study suffers from selective attrition. Most important is that respondents who completed the one-year follow-up experienced less severe depressive and anxiety symptoms at baseline than non-completers, while severity is our outcome measure. We were able to include respondents who only completed the baseline assessment in the multilevel analyses examining the course of anxiety and depression. However, since we aimed at predicting poor clinical outcome at T1 in the following analyses, imputation of missing data was impossible.

A final limitation concerns the generalizability of our findings. Since respondents were recruited from the vicinity of three large cities, people from these highly urbanized regions were overrepresented in our sample. Also, two patient groups are underrepresented in the NESDA study: those who rarely or never visited their general practitioner and therefore could not be approached to take part in this study during the four months of recruitment, and patients who were not fluent in Dutch.

### Clinical implications

An important implication of our study is the necessity to differentiate between several groups of untreated patients. Rost's [16] finding that untreated depression has a poor prognosis should be limited to those people suffering from depression (or anxiety disorder) with unmet needs for care. Our results imply that half of the respondents in the untreated group, those without a self-perceived mental problem or treatment need, make an adequate estimation of their need for care: they reported less severe symptoms at baseline, and had a mostly favorable clinical outcome at one-year follow-up. Patients with a perceived need for care (which was or was not met) had a poorer outcome, and already suffered from a severe depression or anxiety disorder at baseline. However, untreated patients with a depressive disorder who expressed a need for care showed the least improvement, lonely patients and those with a comorbid anxiety disorder in particular. This is the target group Rost [16] is aiming at. Therefore, it is important that primary care workers pay attention to a patient's need for care, Especially, patients with a low social-economical status and little support with some signs of depression or anxiety might be systematically prompted about a possible need for care [29].

The course of anxiety and depression did not differ significantly between untreated patients with a perceived need for care, and those who received treatment. This raises the question whether treatment could have improved clinical outcome in those untreated patients with a need for care. However, these results must be interpreted with caution, as mentioned before. First of all, patients in the treated and non-treated groups were not randomly assigned to their conditions. Instead, distinctions were based on self-selection. Therefore, other factors determining important differences between these groups could account for the absence of differences in clinical outcome. In addition, it may well be the case that without receiving treatment, the now treated persons would have had much higher symptom levels or a poorer course. Apparently, receipt of and need for care are not independent of symptom severity in predicting the outcome of depression and anxiety. Similarly, utilization of professional care appeared to be the strongest predictor of poor outcome in the NEMESIS study, causing symptom severity to lose its significance in the prediction model [29]. It is important to realize that our observational cohort results for treated and non-treated persons cannot be directly interpreted as providing evidence for the effectiveness of treatment. Therefore, it would be of interest to investigate in more detail the differences between patients who do receive treatment, and those who do not although they perceive a need for care, in terms of personality characteristics, a prior history of anxiety and depression etc. Furthermore, this study considers patients to be treated when they confirmed contact with one or more (mental health) care providers for their anxiety or depressive disorder. However, we do not know how intensively they were treated. For instance, it is unknown whether they visited their GP only once, or attended frequently for their mental problem. Clearly, greater understanding is needed in this area.

## Conclusion

Our study identified a considerable number of patients with a current anxiety or depressive disorder and an unmet need for care, who showed the poorest one-year outcome compared to untreated patients without a need for care. Therefore, primary care workers should perhaps pay more attention to these patients, look actively among risk groups (low SES, low social support) for possible cases, explore their possible needs for care and support them in making an informed decision on whether or not to seek further treatment

## Competing interests

The authors declare that they have no competing interests.

## Authors' contributions

IvB and PV participated in the design of the study, performed and interpreted the statistical analyses and were involved in drafting the manuscript. PC and HM have critically revised the manuscript. BP is the principal investigator of the NESDA study, and participated in the design of the study and revising the manuscript. All authors read and approved the final manuscript.

## Pre-publication history

The pre-publication history for this paper can be accessed here:

http://www.biomedcentral.com/1471-244X/10/86/prepub
